# Geographic variations in cervical cancer risk in San Luis Potosí state, Mexico: A spatial statistical approach

**DOI:** 10.1186/s12939-016-0448-z

**Published:** 2016-09-29

**Authors:** Mónica Terán-Hernández, Rebeca Ramis-Prieto, Jaqueline Calderón-Hernández, Carlos Félix Garrocho-Rangel, Juan Campos-Alanís, José Antonio Ávalos-Lozano, Miguel Aguilar-Robledo

**Affiliations:** 1 Geography Graduate Programme, Universidad Nacional Autónoma de México (UNAM), Ciudad de México, México; 2 Department of Environmental Epidemiology and Cancer, National Centre for Epidemiology, Carlos III Institute of Health (ISCIII), C/Monforte de Lemos 5, Pab. 12, 28029 Madrid, Spain; 3 Centre of Applied Research in Environmental and Health, Coordination of Innovation and Application in Science and Technology, Universidad Autónoma de San Luis Potosí (UASLP), Unidad de Posgrado 550 Av. Sierra Leona, Lomas 2ª sección, San Luis Potosí, S.L.P. 78210 Mexico; 4 El Colegio Mexiquense, A.C. Ex-Hacienda Santa Cruz de los Patos, Zinacantepec, CP 51350 Mexico; 5 Faculty of Geography, Universidad Autónoma del Estado de México (UAEM), Cerro de Coatepec s/n Ciudad Universitaria, CP 50110 Toluca, Mexico; 6 Regional Laboratory of Variability, Climate Change and Environmental Risk Assessment, Universidad Autónoma de San Luis Potosí (UASLP), Unidad de Posgrado 550 Av. Sierra Leona, Lomas 2ª sección, San Luis Potosí, S.L.P. 78210 Mexico; 7 Faculty of Social Sciences and Humanities, Universidad Autónoma de San Luis Potosí (UASLP), Campus Oriente, 101-A Av. Industrias, Fracc. Talleres, San Luis Potosí, S.L.P. 78399 Mexico; 8 Universidad Autónoma de San Luis Potosí (UASLP), Campus Poniente, 130 Av. Niño Artillero, Zona Universitaria, San Luis Potosí, S. L. P. 78210 Mexico

**Keywords:** Spatial analysis, Bayesian approach, Cervical cancer, Generalised lineal mixed models, San Luis Potosí, México

## Abstract

**Background:**

Worldwide, Cervical Cancer (CC) is the fourth most common type of cancer and cause of death in women. It is a significant public health problem, especially in low and middle-income/Gross Domestic Product (GDP) countries. In the past decade, several studies of CC have been published, that identify the main modifiable and non-modifiable CC risk factors for Mexican women. However, there are no studies that attempt to explain the residual spatial variation in CC incidence In Mexico, i.e. spatial variation that cannot be ascribed to known, spatially varying risk factors.

**Methods:**

This paper uses a spatial statistical methodology that takes into account spatial variation in socio-economic factors and accessibility to health services, whilst allowing for residual, unexplained spatial variation in risk. To describe residual spatial variations in CC risk, we used generalised linear mixed models (GLMM) with both spatially structured and unstructured random effects, using a Bayesian approach to inference.

**Results:**

The highest risk is concentrated in the southeast, where the Matlapa and Aquismón municipalities register excessive risk, with posterior probabilities greater than 0.8. The lack of coverage of Cervical Cancer-Screening Programme (CCSP) (RR 1.17, 95 % CI 1.12–1.22), Marginalisation Index (RR 1.05, 95 % CI 1.03–1.08), and lack of accessibility to health services (RR 1.01, 95 % CI 1.00–1.03) were significant covariates.

**Conclusions:**

There are substantial differences between municipalities, with high-risk areas mainly in low-resource areas lacking accessibility to health services for CC. Our results clearly indicate the presence of spatial patterns, and the relevance of the spatial analysis for public health intervention. Ignoring the spatial variability means to continue a public policy that does not tackle deficiencies in its national CCSP and to keep disadvantaging and disempowering Mexican women in regard to their health care.

## Background

Cervical cancer (CC) is the fourth most common cancer in women and the seventh overall in the world, affecting 528,000 individuals each year worldwide, with an age-standardised incidence rate (ASR) of 14.0 per 100,000 women. CC is reflected in different geographic distributions, and is the leading cause of mortality among women in the least developed regions, with about 230,158 deaths each year (rate 10.2 per 100,000), as compared with more developed regions that have 35,495 deaths each year (rate 3.3 per 100,000). More than 85 % of the global burden of CC, which is caused by high-risk Human Papillomavirus (HR-HPV), occurs in low and middle-income/Gross domestic product (GDP) countries [1, 2].

In Mexico, CC affects 13,960 women 15 years old or older (ASR 23.3, incidence rate per 100,000) annually. According to the Mexican Women´s Cancer information System (SICAM), the standardised CC mortality rate dropped between 1990 and 2010, from 28.7 to 14.6 per 100,000 women aged 25 and over; SICAM did not register mortality in women aged between 15 and 24 [3, 4]. These high incidences and mortality rates can be considered evidence of existing disparities related to socioeconomic and geographic factors.

The population in Mexico, face problems related to many factors (social, economic, environmental, cultural and access to health care services) the impact of which increases the risk of a range of diseases. The probability of access to prevention programmes, plus appropriate and early treatment, and follow up also impact this risk.

There are adverse modifiable factors related to:

- Lack of drinking water and basic sanitation services.
- Lack of health infrastructure and poor access to what does exist.
- Lack of education and hygiene.

**Fig. 1:**
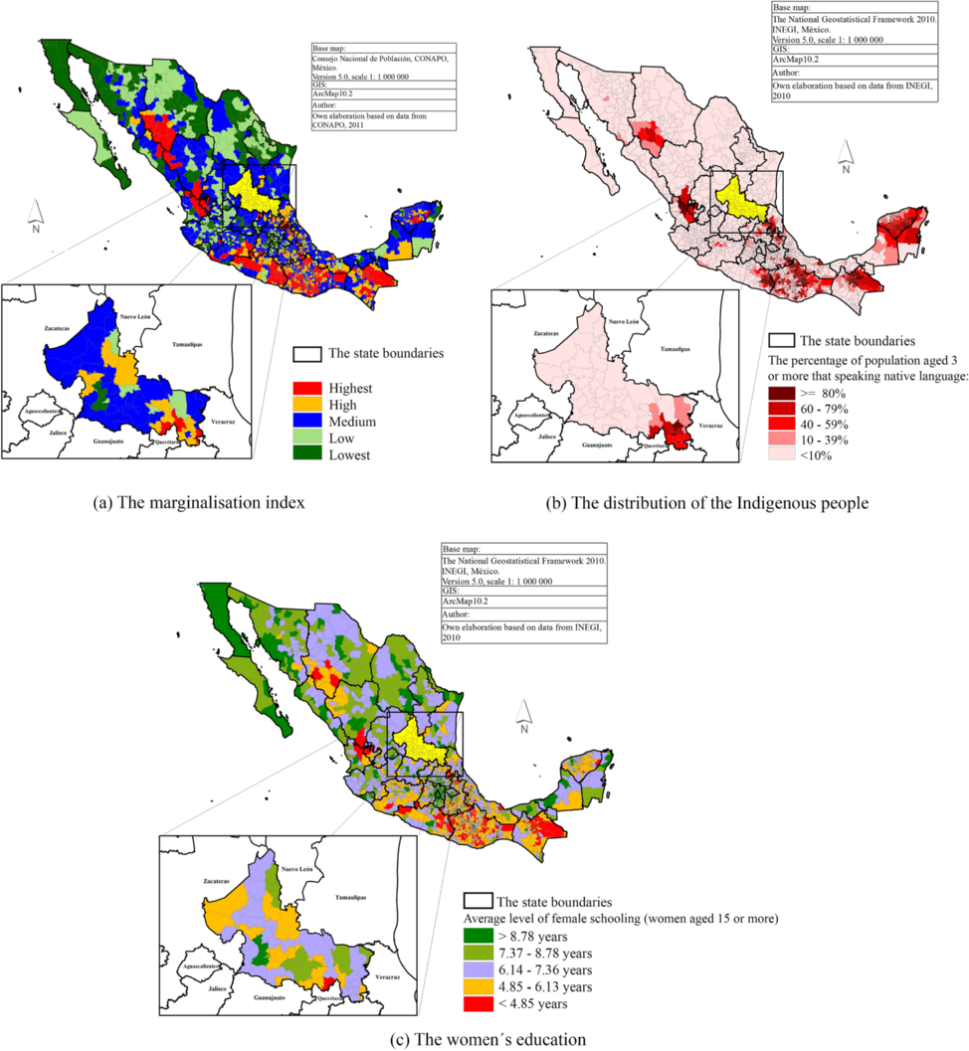
Some indicators of the Mexican population living conditions

**Fig. 2:**
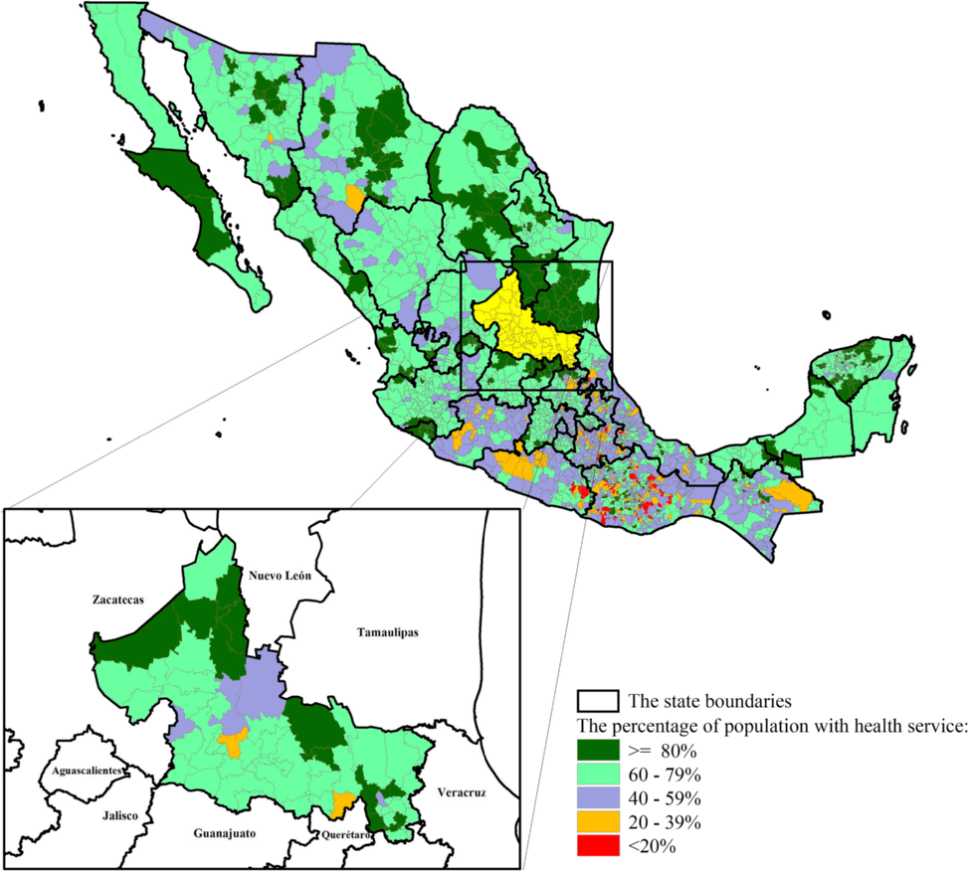
The Distribution of the population with public health system in Mexico, by municipality level

Besides the above mentioned, more than 35.70 % of the Mexican population live under conditions of high marginalisation and poverty. Mainly this relates to those distributed in the states of Veracruz, Puebla, Chiapas, Michoacán, Oaxaca, Guerrero, Hidalgo, San Luis Potosi, Tabasco, Yucatan and Campeche [5]. Figures 1 (in a, b, and c) and 2 give more specific information at a municipal level:

- Figure 1 shows the distribution of (a) the marginalisation Index, (b) Indigenous people, and (c) women’s education.
- Figure 2 provides the distribution of the population with public health system in México City.

The incidence of CC is higher in states with high marginalisation, where women have little or no access to early detection and treatment. For example, in San Luis Potosí (SLP) state, which ranks 8th in CC mortality risk in the country [3] and also ranks 7th in deprivation and lack of basic socioeconomic resources [5], CC incidence is 52.80 per 100,000 (age-standardised incidence). These rankings show that CC still remains a public health problem and different strategies need to be undertaken and improved [6].

The highest percentage of the population with high marginalisation and poverty in the state of San Luis Potosi, is concentrated to the southeast and some municipalities in the north and northwest (Fig. 1a).

In another published study it is reported that in the south-east, 62.5 % of the working population receives an income of less than $1.25 (USD) a day, a high percentage does not have piped water 76.6 %; 100 % no drainage; 90 % have soil floors; 30 % have no separate kitchen and 96.6 % live in overcrowded conditions (four to five members sleep in one room), all those are factors that generate scenarios of greater vulnerability [7]. Moreover, the south east region also has more than 80 % of the state´s indigenous people living there (Fig. 1b).

In the past decade, several studies of CC, identifying the main modifiable and non-modifiable CC risk factors for Mexican women, have been published. Some of the modifiable factors are:

- sexual relations before 20 years of age (OR = 2.60, 95 % CI: 1.75–3.95) and more than 4 childbirths (OR = 4.25, 95 % CI: 2.15–8.38) which are considered as cofactor in the development of cervical neoplasia, particularly for women infected with HR-HPV.
- Women with lower than 6 years of schooling (OR = 3.24, 95 % CI: 1.97–5.33).
- Illiteracy (OR = 4.75, 95 % CI: 2.94–7.69).
- Lack of access to a health care system (OR = 5.72, 95 % CI: 3.28–9.99).

As previously mentioned, all of these are modifiable [6, 8, 9]. Furthermore, women living in rural areas have 3.07-fold higher CC mortality risk compared to women with an urban residence. Some Mexican states also show a statistically significant difference in mortality from the national average, with women residing in the southern part of the country being at greater risk of dying from CC [3].

However, in Mexico there are no studies that attempt to explain the residual spatial variation in CC incidence, i.e. spatial variation that cannot be ascribed to known, spatially varying risk factors.

Given the above evidence, efforts to reduce CC begin with an understanding of incidence, mortality and prevalent geographical patterns. The aim of this paper is to analyse the geographical pattern of CC incidence in SLP state, using spatial statistical methodology that takes into account spatial variation in socio-economic factors and the accessibility to health services, whilst allowing for residual, unexplained spatial variation in risk. Our results are relevant to the implementation of the Mexican cervical cancer screening programme (CCSP) and could improve the effectiveness of decision-making in relation to preventive interventions.

## Methods

### Study-design

**Fig. 3:**
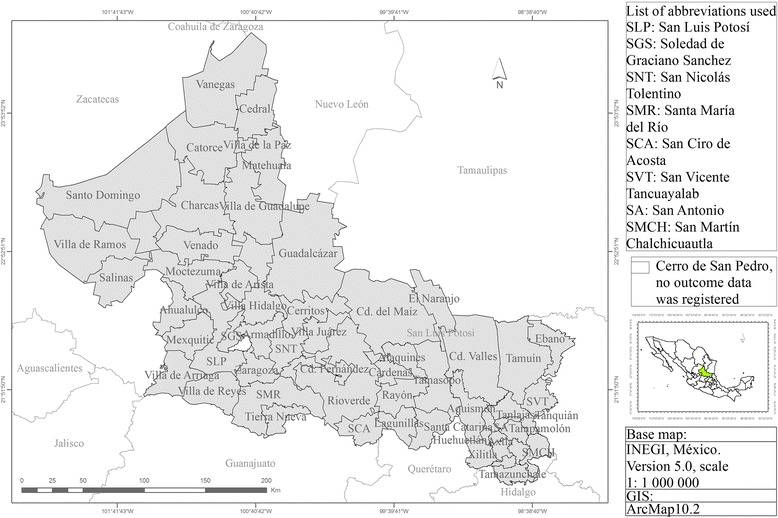
San Luis Potosi, SLP State, Mexico: Municipality division

This was a geographical study based on CC incidence data for each of the 57 municipalities of SLP state in Mexico, which is located in the North-Central area of Mexico with latitude 21° 10´ to 24° 29´ north, longitude 98° 20´ to 102° 18´ west. One municipality was not included in the study because no outcome data was registered during the study period (Fig. 3).

We combined two data sets for the spatial analysis. For the first of these, CC case-counts were drawn from the records in the public health system of the Epidemiology and Statistics Department, State Health Services (SSA) of SLP state over the study-period 2005–2010 for the 57 SLP municipalities; codes C53, D06 under the International Classification of Diseases-10 (ICD-10). The operation of the Mexican Health Service is under the Ministry of Health; the service is integrated between public and private institutions.

For the second data set, population data was extracted from the 2010 Mexican census, carried out by the National Institute of Statistics and Geography [10], which also provided socio-demographic information for each municipality (count data aggregated by municipal level).

We included several socioeconomic covariates in our analysis. The chosen socioeconomic covariates fall into two categories. Those related to individual women are expressed as percentage amongst women aged 15 years old or more, namely: illiterate; unemployed; single females (SF); born in SLP; head of household and lacking social healthcare protection. Other covariates are descriptors of the municipalities themselves, namely: marginalisation index (MI) which is an overall measurement of deprivation and lack of basic socioeconomic resources in the Mexican population, as well as urban inequality [5].

In this study, we calculated the following three covariates for the model: the Coverage of Cervical cancer screening programme (CCSP); the Positive screening Index and the Index of Accessibility to health services.

The Coverage *C* and The Positive screening Index *PI* are defined below [11]:

$$ {C}_i = \frac{{\mathrm{W}}_{\mathrm{i}}\ }{O_{\mathrm{i}}} \times 100 $$

where *i* = 1,2,…, N

C is the percentage of women aged 15 or more with pap smear, *W*_*i*_ represents the total female population aged 15 or more with a pap smear in the last year in municipality *i* and *O* represents the total risk population in municipality *i*.

$$ P{I}_i = \frac{A_i}{w_i} \times 100 $$

where *i* = 1,2,…, N

*PI*_*i*_ is used to measure the detection of abnormal or cancerous cells, $A$ represents the total of positive pap smears to abnormal or cancerous cells in women aged 15 years of age or older in the last year in municipality *i* and $w$ accounts to women aged 15 years of age or older with a pap smear in the last year in municipality *i*.

Finally, the Index of Accessibility to health services (IA). A gravity model as an indicator of the territorial dimension is used to estimate local and global accessibility, potential access to health services [12]. The work was carried out on a scale of territorial disaggregation of Basic Geostatistical Areas (BGA): 6829 localities are included from 58 municipalities and Medical Unit (MU): 300 medical units, considering the place where the MU is located.

This article uses a method that complies with the two fundamental premises of our proposal: it is simple and financially feasible. There are other more precise methods e.g. the 2SFCA and its derivatives [13–17]; and more accurate processes e.g. measure the distance through the network of roads and highways, using mathematical functions of accessibility [18–21]. However, these methods are not feasible in our study area for technical reasons (e.g. mathematical abilities and statistics of planning agencies) and the financial situations of SLP state governments.

The *IA* is defined below:

$$ IA = {\displaystyle \sum_j}\frac{S_j}{\frac{O}{d_{ij}^b}} $$

*S*_*j*_ represents the magnitude of available services per UM *j*, daily availability of medical appointments is 3000 per doctor according to the type of UM; $O$ represents the total of users in the study area, there are 925, 688 women aged 15 or more as potential users in SLP state; *d*_*ij*_ is the distance between the place of residence-municipality *i* and the place where health service is located, medical unit *j*; and *b* represents the spatial behaviour of users with respect to distance.

Hence:

$$ {d}_{ij}=\sqrt{{\left({x}_1-{x}_2\right)}^2+{\left({y}_1-{y}_2\right)}^2}/3000 $$

Where:

*x*_1_ coordinate *x* from origin *i*

*x*_2_ coordinate *x* from the place where medical unit is located *j*

*y*_1_ coordinate *y* from origin *i*

*y*_2_ coordinate *y* from the place where medical unit is located *j*

It should be noted that we did take into account the road network to calculate distances, especially in the southeast of SLP state, which is very rugged. Studies of correlation between linear distance and distance in kilometres on roads and paths, with data from the Communications and Transportation Secretariat (CTS) showed the correlations were higher than 0.70, the same is reported in other recent works [22]. We carried out simulation exercises to test various locational solutions, using the linear distance and the one related to highways and roads network. The results were not very different in terms of improving population accessibility however the option of using the linear distance is much more practical and feasible for planners in the public sector [12, 23].

The network analyst and computations were carried out using ArcMap 10.2.

### Statistical models

To describe variations in CC risk, we used generalised linear mixed models (GLMM) with spatially structured and unstructured random effects [24–27]. Specifically, we assumed that the number of disease cases *Y*_*i*_ in municipality *i* = 1, …, 57 is Poisson distributed with mean *μ*_*i*_, conditional on measured covariates and unmeasured random effects, hence;

$$ {Y}_i\sim Po\left({\mu}_i\right),\ {\mu}_i = {E}_i{\theta}_i\kern2.75em i:1,\dots, 57 $$

In this equation, *θ*_*i*_ is the unknown risk relative to the whole study-area, and *E*_*i*_ is the expected number of cases, adjusted for variation between municipalities in the sizes, *N*_*i*_, of the population at risk, hence;

$$ {E}_i={N}_i\frac{{\displaystyle {\sum}_i}{y}_i}{{\displaystyle {\sum}_i}{N}_i}={N}_ip $$

Where *p* is the estimated average risk over the whole of SLP state.

The relative risk is θ_i_, that is the estimation of the standardise incidence ratio (SIR) by the conditional autoregressive model (CAR). We cannot use SIR because we estimate it, hence using relative risk, following Clayton’s paper [25].

The logarithm of the relative risk, log (*θ*_*i*_), in each spatial unit is then decomposed into a deterministic part explained by a set of covariates *X*_*ik*_ with associated regression parameters *β*_*k*_ and an unobserved stochastic part, that in turn has two components: an unstructured component, *U*_*i*_ and a spatially structured component, ${S}_i$ [26].

For the stochastic part of the model, the *U*_*i*_, are normally distributed and independent, with mean zero and variance *σ*^2^, whilst the *S*_*i*_ are also normally distributed, but follow a spatially dependent intrinsic conditional autoregressive model (CAR) [26], leading to the log-linear specification;

$$ \log \left({\theta}_i\right)={\displaystyle \sum_{k=1}^m}{\beta}_k{X}_{i,k}+{U}_i+{S}_i $$

In the CAR model, the distribution of each *S*_*i*_, conditional on all of the *S*_*j*_*i* ≠ *j*, is normal with a mean equal to the average of the *S*_*j*_*´s* for municipalities deemed to be neighbours of the *i* th municipality, and variance *τ*^2^/*n*_*i*_ where *n*_*i*_ is the numbers of neighbours of the *i* th municipality. We defined the neighbours of the *i* th municipality to be those that shared a common boundary with the *i* th municipality.

To estimate the model parameters, we used a Bayesian approach with normal priors for the regression parameters *β*_*k*_ and half-normal priors with variance *τ*^2^/*n*_*i*_ for standard deviations *σ* [26].

The computations were carried out using Integrated Nested Laplace Approximations as implemented in the R-INLA package within the open source R software environment [27, 28]. We drew maps using ArcGIS 10.2.

The output from the analysis includes posterior distributions for the model parameters and for the random effects, enabling estimation of municipality-level risk with or without adjustment for covariate effects. To detect municipalities with higher than average risk with adjustment for covariates, we used the criterion of a posterior probability that RR > 1, we applied Richardson´s criterion [29], which recommends those RR with probability above 0.8 to be considered statistically significant.

To compare specific models within the overall model class, we used the Deviance Information Criterion (DIC), where smaller values of DIC indicate a better model fitting [30]. This proved to be useful tool to compare models and to challenge the model under study.

## Results

Our results show that in the study period 2005–2010, 1,586 cases of CC were diagnosed in the SLP state. The Age-Standardised Rate (ASR) was 52.8 for 100,000 women 15 years of age or older.

Table 1 shows the descriptive statistics for population and CC cases in the region during the study period.

**Table 1:** Summaries of women population and cervical cancer (CC) cases

	Total	Mean	Median	Standard deviation	Min.	Max.	Number and percentage (%) of municipalities with zero count
Risk population	924296	16215.7	6872	39,662.7	1621	291,154	0
Observed CC cases	1586	28	14	47.59	0	309	1(1.7 %)^a^
Expected CC cases	2697	47	21.3	112	5.7	834.3	0

^a^Armadillo de los Infantes municipality

Table 2 shows the ASR and proportions.

**Table 2:** The Age-Standardised Rate (ASR) and proportions per 100,000

Women aged group	ASR	%
15–24 years of age	7.62	4
25–34 years of age	36.37	16
35–44 years of age	64.29	22
45–64 years of age	96.02	44
65 years of age or older	89.02	14
All aged group	52.80	100

The coverage of CCSP in the state’s target population is around a 13.9 %. The mean of positivity to cervical intraepithelial neoplasia grade III (CIN 3) and CC in advanced stages of illness (CIE-10 D06 and C53) was 14.03 %, with variations in its distribution between municipalities, CC was concentrated in the southeast of the state.

**Fig. 4:**
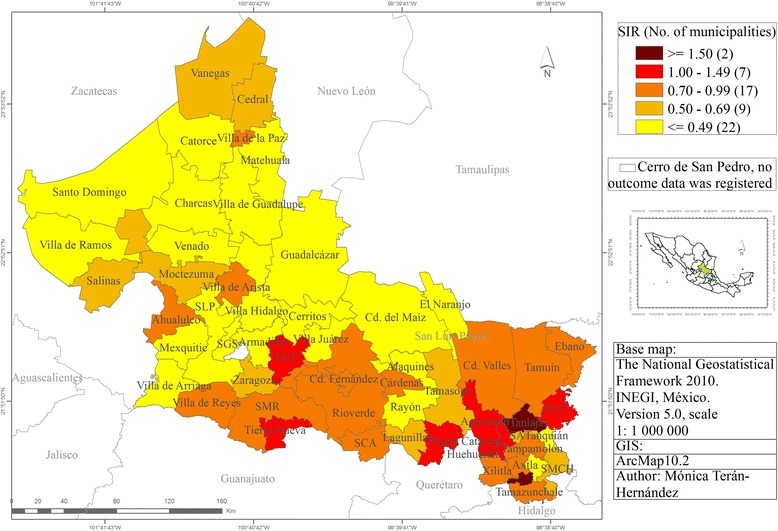
The observed standardised Incidence Ratio of Cervical Cancer, SLP state, Mexico from 2005 to 2010

Figure 4 shows the observed Standardised incidence ratio (SIR) values for each municipality from 2005 to 2010. The municipalities with the highest SIR values > = 1.50 -Tanlajás and Matlapa- are located in the southeast of the state. The municipalities with the lowest SIR values are located in the west and north; only Villa de la Paz (0.87) has a high SIR value. We identify that Villa de Arista and Ahualulco have high values compared to the midland state municipalities.

**Fig. 5:**
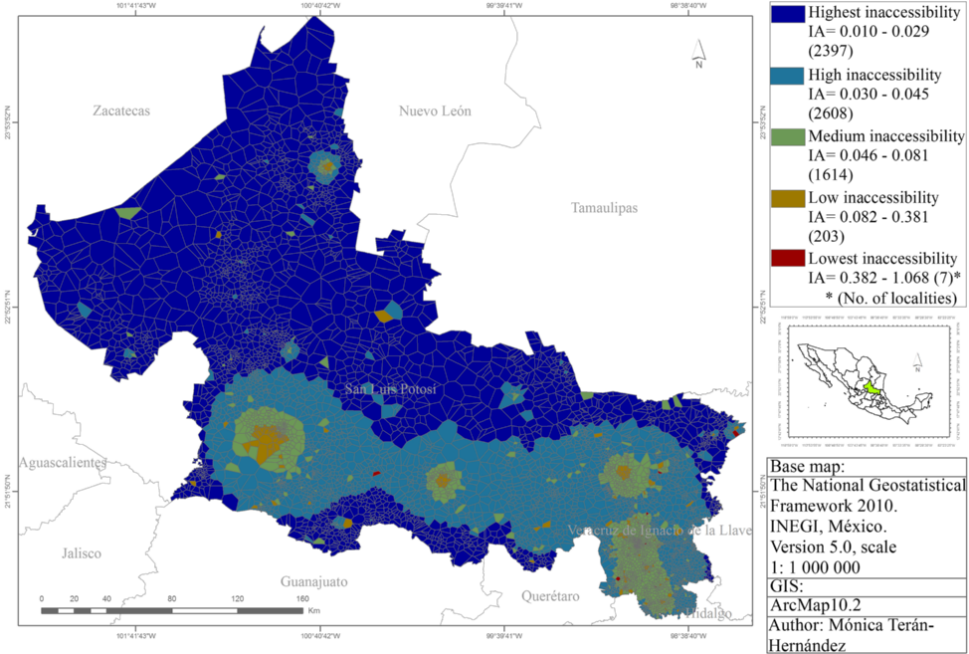
The Accessibility to prevention, early detection, diagnosis and management of Cervical Cancer, SLP state, Mexico

Figure 5 shows the accessibility of public health services to Cervical Cancer prevention, diagnosis and treatment, as an indicator of the territorial dimension of health.

Table 3 presents the results from the GLMM models where models number 2 (DIC 343.69) and 4 (DIC 344.31) are the better fitting model. These models show the smallest DIC and it should be noted that they did not show differences in the patterns, which is why we focused on the number 4, model with both spatially structured and unstructured random effects.

**Table 3:** Better fitting model: Analyses results of Models

		mod1 *U*_*i*_, *S*_*i*_≡0	mod2 *S*_*i*_≡0	mod3 *U*_*i*_ ≡0	mod4 *U*_*i*_, *S*_*i*_ ≠ 0
*β*_1_ Unemployed	*μ*	−0.014	−0.033	−0.022	−0.032
	*sd*	0.009	0.014	0.011	0.014
*β*_2_ Single female (SF)	*μ*	0.033	0.041	0.033	0.44
	*sd*	0.010	0.015	0.014	0.015
*β*_3_ Marginalisation Index (MI)	*μ*	0.043	0.062	0.041	0.052
	*sd*	0.006	0.013	0.009	0.010
*β*_4_ Positive screening Index (PI)	*μ*	0.043	0.028	0.018	0.028
	*sd*	0.009	0.015	0.014	0.015
*β*_5_ Coverage (C)	*μ*	0.143	0.171	0.146	0.154
	*sd*	0.014	0.024	0.020	0.022
*β*_6_ Index of accessibility to health services (IA)	*μ*	0.020	0.014	0.017	0.013
	*sd*	0.006	0.009	0.008	0.009
DIC		372.72	343.69	348.73	344.31

Deviance Information Criterion (DIC), posterior mean (μ) and posterior standard deviation (*sd*) of the fixed effects for the four GLMM models, models number 2 and 4 fitted better. Where; *S*_*i*_ is the spatially structured and *U*_*i*_ is the unstructured random effects.

The fixed effects (*α*, *β*_1_, …, *β*_6_) estimated by model number 4 are presented in Table 4. If these effects are exponentiated, they can be interpreted as relative risk (RR). Where, there is an increase in the risk of CC per increase in: single female percentage (RR 1.04, 95 % CI 1.01–1.04); Marginalisation Index (RR 1.05, 95 % CI 1.03–1.08); Positive screening index (RR 1.02, 95 % CI 1.0–1.05); lack coverage of CCSP (RR 1.17, 95 % CI 1.12–1.22); and lack of accessibility to health services (RR 1.01, 95 % CI 1.00–1.03). Thus, there is an increase in the risk of CC per decrease in working women percentage (RR 0.97, 95 % CI 0.94–0.99).

**Table 4:** Results from GLMM-model number4

Fixed effects		mean ($\mu$)	$sd$	2.5 %	50 %	97.5 %
*α* α		−2.122	1.185	−4.393	−2.143	0.268
*β*_1_	Unemployed	−0.032	0.014	−0.062	−0.032	−0.006
*β*_2_	Single female (SF)	0.044	0.015	0.014	0.044	0.075
*β*_3_	Marginalisation Index (MI)	0.052	0.010	0.032	0.052	0.074
*β*_4_	Positive screening Index (PI)	0.023	0.015	−0.008	0.023	0.053
*β*_5_	Coverage (C)	0.154	0.022	0.111	0.153	0.199
*β*_6_	Index of Accessibility to health services (IA)	0.013	0.009	−0.005	0.013	0.032

Summary statistics posterior mean ($\mu$); posterior standard deviation ($sd$) and posterior 95 % credible interval

**Fig. 6:**
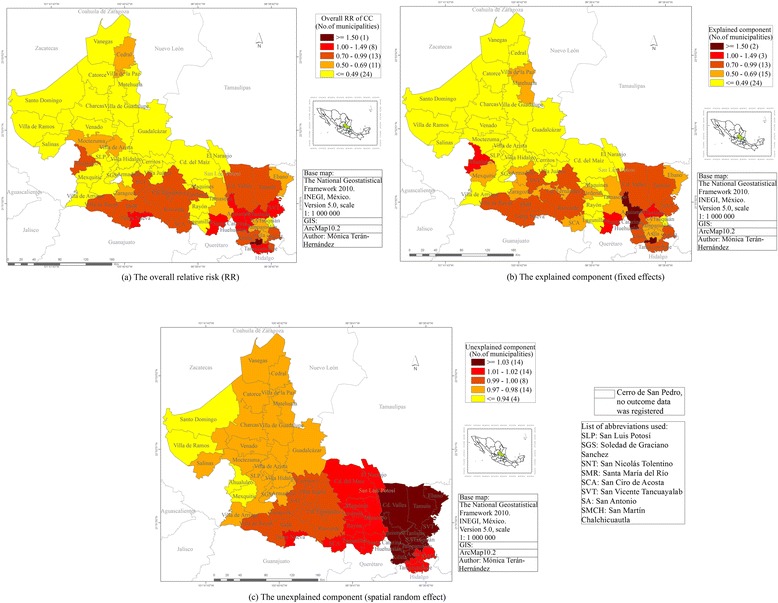
Risk-maps of Cervical Cancer from 2005 to 2010

After adjusting the GLMM model, the smoothed relative risk varies from location in respect of initial risk and, three risk-maps are presented:

Figure 6a provides an overall relative risk (RR). The municipality of Matlapa shows the highest RR, RR 1.80 (95 % CI 1.37–2.27). Some other municipalities with high RR are: Tierra Nueva RR 1.15 (95 % CI 0.75–1.67) in the south; and Aquismón RR 1.42 (95 % CI 1.11–1.17), Tanlajás RR 1.24 (95 % CI 0.92–1.66), Santa Catarina RR 1.14 (95 % CI 0.77–1.63), Tancanhuitz RR 1.07 (95 % CI 0.77–1.41), Huehuetlán RR 1.03 (95 % CI 0.70–1.43), SVT RR 1.07 (95 % CI 0.75–1.47) and Tamazunchale RR 1.0 (95 % CI 0.82–1.19) in the south-east. The municipalities with the lowest risk are: Cedral RR 0.57 (95 % CI 0.37–0.83), Villa de la Paz RR 0.68 (95 % CI 0.43–1.03), Ahualulco RR 0.93 (95 % CI 0.64–1.25) and Moctezuma RR 0.51 (95 % CI 0.35–0.72), Tamasopo RR 0.64 (95 % CI 0.47–0.83), Rayón RR 0.37 (95 % CI 0.22–0.54) and Lagunillas RR 0.44 (95 % CI 0.28–0.64).

Figure 6b shows a map of the explained component (fixed effects). In this risk-map we identify two municipalities with RR higher than 1.50, Matlapa with RR 2.13 (95 % CI 1.78–2.52) and Aquismón RR 1.57 (95 % CI 1.29–1.87). The municipalities with RR higher than the state of SLP are: Tanlajás RR 1.02 (95 % CI 0.92–1.11), Santa Catarina RR 1.07 (95 % CI 0.90–1.26) and Ahualulco RR 1.05 (95 % CI 0.89–1.23). All these municipalities are located in the southeast of the state except the last one, Ahualulco, which shows a different pattern to its neighbours in the centre of the state.

The unexplained component (spatial random effect) is included in Fig. 6c, where more details are shown, for instance the patterns of the highest risk in the southeast (1.03–1.08) and lowest risk in the northwest (0.94–0.97). In the southeast of the state, Tamazunchale 1.02 (95 % CI 0.89–1.18), SMCH 1.01 (95 % CI 0.88–1.15), Tampacán 1.02 (95 % CI 0.91–1.17) and Matlapa 1.02 (95 % CI 0.91–1.19) show a RR pattern unlike its neighbours.

**Fig. 7:**
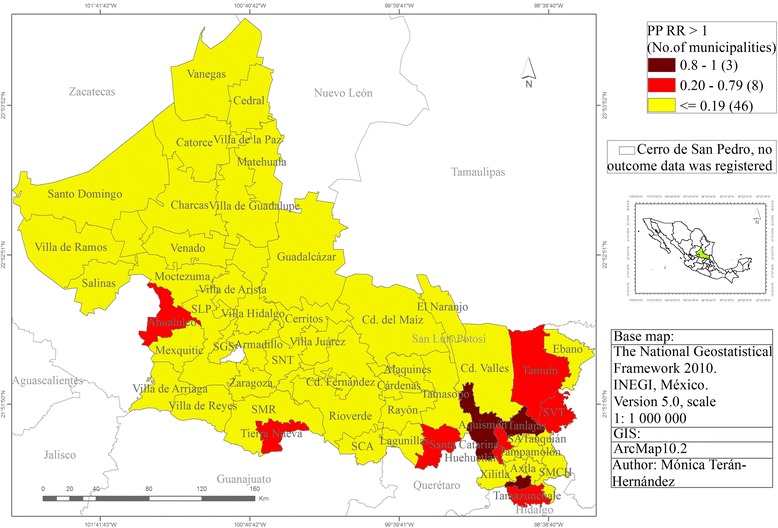
The distribution of the posterior probability of RR-Cervical Cancer

Finally, Fig. 7 shows the distribution of the posterior probability of RR. Three municipalities, Aquismón (1.0), Matlapa (1.0) and Tanlajás (0.92) registered excess risk, with posterior probabilities greater than 0.8. All of these are located in southeast part of SLP state.

## Discussion

Our results confirm that mapping of the RR of CC shows substantial spatial variation throughout SLP state, even after adjusting for known and hypothesized risk-factors that are available to us as spatially referenced covariates. The highest risk is concentrated in the southeast, where the Matlapa and Aquismón municipalities, are located. The municipalities of Villa de la Paz to the north, Tierra Nueva to the south and Ahualulco to the northwest attract our attention because of their similar pattern to those of the southeast. The lowest RR values are located in the north and in the centre of the state. Furthermore, between the areas of the highest and lowest risk there are socioeconomic inequalities and differences in accessibility to health services. There is a high in-accessibility to CC prevention in SLP state, mainly in the southeast area.

The use of a method such as the 2SFCA (and its many variants) can be marginally more accurate than the method used in our article, however it is much less applicable in SLP state which lags behind others in the field of governmental planning and innovation [31, 32].

One of the most important problems when applying the 2SFCA methods (and their variants) in non-advanced regions of emerging countries, such as SLP [31–33], is the selection of a mathematical function that adequately represents spatial accessibility. This is a complex problem, even for experienced researchers, however In SLP, the technical capacity of state and local government´s planners is very low and finding an acceptable spatial accessibility feature is probably beyond their capabilities. In addition, the rotation of personnel in the public sector is very high, registering changes of officials every three or six years, which seriously limits the formation of specialists [31, 32].

Even more so, the indicator of accessibility is only a guide to orientate spatial planning of health units and not an accurate measurement. It does not take in to account for example, women’s perceptions as potential users, on questions such as; what is perceived as safer? Which means less effort or risk? Moreover, it should also be noted that many areas of SLP are areas dominated by organised crime.

Our results show that the highest weight in explaining the CC incidence risk pattern was attributable to coverage of CCSP, marginalisation index and index of accessibility to health services.

The coverage of the CCSP in the state’s target population is very low, particularly in women younger than 25. This calls attention to the significant problem of CC in the SLP state where advanced stages of CC illness are concentrated in the southeast of the state. However there are no other studies in Mexico concerning the distribution of CC’s incidence using a spatial analysis methodology.

Like this study, studies executed in other countries have shown spatial variation in the geographical pattern of CC incidence associated with the distribution of socioeconomic factors [34, 35].

For instance, Lorenzo et al. [34] found that spatial variation of RR of CC was associated with the territorial distribution of socioeconomic factors and with lifestyles. Their results showed two areas with excess risk in Cuba, to the east and far west of the island (CAR-smoothed RR values 1.2 and 2.01) and the lowest risk was shown in the Midwest of that country. This corroborates the findings of this study, that the municipalities with excess risk and extreme RR values can be geographically contiguous. Our results showed a specific spatial pattern for the excess of risk for CC incidence.

In another study, Cheng et al. [35] also revealed a spatially varying regression coefficient between CC incidence and socioeconomic covariates across England, where low social status was the most significant factor related to CC incidence rate. The effect of and contribution from the low social status variable, varied between 0.07 and 4.40 times across England, with the greater contribution from this variable in the south and northeast. These researchers indicated that this might be related to population structure. In our study, the area with excess risk and high RR was the southeast, an area characterised by high marginalisation and poverty.

According to another study, spatial variation in the relationship between the incidence of CC and socioeconomic indicators showed that socioeconomic status had a greater effect over the incidence of CC in some locations than in others. That is to say, the dynamic of the illness was highly correlated to the context under which women lived [36]. In our results, the RR of CC showed substantial spatial variation over the state of SLP, some municipalities with similar RR tended to occupy adjacent locations on the map and some municipalities with high RR tended to be located next to low ones, such as Ahualulco, Tamazunchale and Tierra Nueva. One interpretation of the previous information could be that variations in territorial distribution lead to a stratification of advantages or disadvantages for women in terms of decisions for health care across SLP state. The affecting factor seems to be more related to access to health care services.

About that, WHO [37] states that the place where we live affects our health and our chances of having a prosperous life. This recognition led to the United Nations Development Programme, UNDP to emphasize local human development, which is to recognize that the place where one lives, conditions or sometimes determines factors such as; when people cannot migrate, plus welfare levels, as location facilitates or limits our access to various resources that make possible our development as human beings. Although the place of residence is not destined, it does represent a set of locational effects that limit or facilitate our development.

WHO recognises that the deprived health of the poor, the social gradient in health within a locality, region, state, country and important health inequalities are caused by the unequal distribution of power, income, goods and services that are affecting living conditions [37]. Secondly that there will often be broad regional processes, whether social, economic or environmental, which ensure that the disease incidence in one small area is similar to the one in neighbouring areas. For example, if this wider region has quite uniform levels of poverty, we can expect rates of some diseases to be higher than in neighbouring small areas that make up the region, like the south and southeast region in our study. It is possible that there exists a socio-spatial determination [38].

The spatial variation in CC’s distribution pattern in the SLP state is associated with the differences in the covariates’ territorial distribution: unemployment, single female, marginalisation, coverage of CCPS and accessibility to medical attention units. These are key components of the women´s living conditions. Cheng et al. demonstrated just the same pattern by showing a locally varying relation between CC incidence and low social status, including unemployment [35]. Unemployed women use health services less frequently or they do not use them at all, which could explain the low opportunity of timely prevention and detection of the illness.

Furthermore, Tirado’s et al. [9] case–control study pointed out that a low socioeconomic level (RMp = 10.8) is a factor associated with increasing the probability of developing CC in Mexican women. In this regard, although six years of education could empower women in terms of decision-making, regarding health and job opportunities, 69 % of Mexican women who died of CC had no formal education [39].

Other studies report that the southeast of the SLP state is characterised by high marginalisation and poverty [7]. This marginalisation index combines nine variables that show some degree of correlation and a tendency to concentrate in non-urban zones, where marginalisation levels are usually higher; these zones are characterised by unemployment and the lowest education level [5].

Low educational achievements limit access to health services and therefore to early detection of illness, such as early detection of CC through the Pap smear (actually HPV DNA screening-testing), and treatment when the disease is in early stages [39]. In relation to this, if a health need is not recognized, the motivation to meet this need is therefore non-existent and as a result, the process of seeking health care is not initiated. This situation contrasts with, high educational achievements which contribute directly to better health along with the possibility of participating wholly in a productive society and utilising health care services. Employment and stable job opportunities could also improve the health of women by guaranteeing access to health services [40]. This should be taken into account so that CC preventive programmes are directed to this high-risk area particularly in frequency, follow-up and control of the actions to be taken. Most importantly, distribution of health service resources should be related consistently to the distribution of CC risk. As such, the programme must consider the spatial dynamics of this health risk.

With respect to single females, our results show that this covariate was significant to the distribution of the RR of CC. In the Cheng et al. [35] study, it showed that the proportion of single females is part of the set of socioeconomic covariates used in the Townsend index that accounts for socioeconomic conditions and, low social status of population contributing to CC incidence with spatial differences. One study in Low-Income Countries showed that being single was a variable significantly associated with a reduced likelihood of a pap smear being taken [41]. This suggests that single female status might be related to a lower possibility of obtaining a pap smear and in turn, the early detection of VPH-AR or early stages of CC illness.

In addition, one of the most important factors to decrease CC incidence is an increase in prevention strategies and early detection. Our results show spatial variation in the relation between incidence and coverage of CCSP across SLP state. The aim of the screening programme is to detect abnormal or cancerous cells at an early stage of the disease, and can increase the chances of detecting cancerous cells sufficiently early to lower the incidence rate and thus, the likelihood of survival may be increased [42, 43]. However, some other factors could be impeding women´s access to health services such as: lack of basic socioeconomic resources, location of the medical attention unit, clinic service hours, costs, migration or change of residence, and organised detection programmes not existing. Also cultural barriers e.g. for Indigenous women or not indigenous.

The population in south east of SLP state, registers a high presence of indigenous women (145,860 total of indigenous women), which is distributed in the 20 municipalities of the southeast area [1]. However difficulties exist in the recording of health indicators (morbidity and mortality). In addition, socioeconomic status, location, and if the woman presenting to the CCSP is indigenous are not recorded. Therefore in the Mexican health service there is no official data for CC in indigenous women.

A contribution of this study, from the perspective of health geography is that it provides a solid basis for making clear the need to support a proposal for improvement: namely, there is a need to have a database record that includes the socioeconomic conditions of the patients, pinpointing where they live, their behaviour, knowledge of prevention, and determinate cultural characteristics, for CC.

One of the factors that makes this theme a complex research area is that although there are negative cultural factor influences within the indigenous population regarding submitting to the pap smear test, in practice it is seen to be a general resistance regardless of whether the individual is indigenous or not.

Our data suggests that a very high proportion of the target population (73.29 %) from SLP state have low accessibility to public service medical attention units (SPSSA). Women who live in municipalities in the northwest and southeast of SLP have very low accessibility to a CC prevention programme or early detection and management of patients with CC (treatment and control services, such as oncomedicine, oncosurgery, radiotherapy and chemotherapy). The only medical attention unit certified as an oncological centre and where all dysplasia cases are referred to is the Hospital Central (Dr. Ignacio Morones Prieto), which is located in the metropolitan area of SLP city and ranks 5th according to the index of accessibility calculated in our study. For most of the women inhabiting the inner SLP states, far removed from the state capital, this hospital is not a viable option for early detection and treatment, before the illness evolves to advanced stages. As demonstrated, CC has a high chance of cure if detected early and treated accordingly [1].

The SLP municipalities in the southeast and north are disadvantaged regarding the equipment for providing prevention, early detection, treatment and control of all positive cases detected early, for the dysplasia process. Therefore, the medical units are unable to meet the demand generated in their respective areas of influence, for instance, in the southeast, where our study found the highest concentration of municipalities with high risk. As Garrocho and Campos [12] and Hernández et al. [44] point out, the distribution and localisation of satisfactory health services represents a condition of advantages and disadvantages to users and that is why it is a key determinant in the population’s health-sickness process.

A high level of CCSP has been the key element in decreasing CC incidence and mortality rates in countries with well-established detection programmes [45]. High levels of coverage of eligible women have principally been achieved in developed countries, for example, the UK´s CC screening programme reported that CC can be prevented by about 75 % provided that women periodically attend screening [35]. Finland is another example of a country with a well organised screening programme, where 72 % of women receive invitations to participate in the Pap screening [3].

In Mexico, as reported by the Ministry of Health, Pap coverage is less than 25 %. One study indicates that the use of HPV testing in combination with a Pap smear could be a cost-effective alternative for CC screening, and should focus on regions with low levels of Pap test coverage, a lack of health care infrastructure and rural and economically disadvantaged communities [3]. Currently, having recognised the poor effectiveness of the Mexican cytology programme, the governmental “Seguro Popular” (a health insurance for low-income citizens) recently introduced an alternative CCSP based on HR- HPV testing for affiliated women aged 35–64 years but it does not cover women younger than 35 years of age [6].

Screening younger women is an issue to be noted. In the UK the number of cases with Cervical Intra-epithelial Neoplasia (CIN3) has increased for women aged between 20 and 24 years because of trends in sexual behaviour. According to the National Health Service (NHS), increasing numbers of young females are becoming sexually active when they are still in their mid-teens and there is a poor use of cervical cancer screening resources [35].

According to our Mexico results, women younger than 25 years old had fewer screening opportunities for timely CC detection. The Age-Standardised Rate (ASR) of CC for younger than 25 years old was 7.62. Allen-Leigh et al. [46] showed that in 2012, 31.2 % of 15–19 years old adolescent Mexican women had undergone their sexual debut. A high percentage of these adolescents (52.2 %) reported not using a condom at their last sexual intercourse, and women in rural areas reported a lower percentage of using a condom.

Note that sexual relations before 20 years of age is a modifying or avoiding risk factor [6, 9]. It explains the transcendence of coverage of CCSP for this population group in Mexico and also that sex education needs to improve in local schools, teaching mid-teen pupils about protective sex, because one key factor to modify or avoid CC risk, is the sexually transmitted HPV infection.

The contribution of socioeconomic factors to CC incidence is clear but it is also evident that in order to achieve a significant decrease in mortality rates, there is a need for a geo-referenced database system of CC cases, with full territorial coverage to allocate resources to make spatially accessible and equitable prevention services that could eventually reduce CC mortality rates. Unfortunately, the current Mexican CCSP organization does not consider the spatial variation of CC incidence.

Expanding the coverage of the CCSP, taking into consideration the spatial variation (i.e. spatial variation that cannot be ascribed to known, spatially varying risk-factors) could therefore improve health education and promote strategies for CC prevention, early detection and management, aimed at increasing women´s knowledge, thereby reducing CC incidence and mortality in SLP and other Mexican states.

The type of medical attention unit (first, second, or third) depends on the size of the population. Resources assignment is through each jurisdiction. It is obvious that there is no consistency between the localisation of Health services and the needs of population in term of the spatiality of this health issue (CC). Spatiality has two key elements: determinations (economic, political, ideological, etc.) and existing factors i.e. accessibility [47].

On the other hand, there is a territorial reality which makes decisions making complex as far as population health care and needs are concerned. There is reflected in many places with migrating and disperse population: a challenge for public policies. It is important to point out that the highest percentage of population is concentrated in urban areas but living in urban area does not guarantee the absence of marginalisation.

There are many observable deficiencies in the current CCSP coverage capacity. Accessibility to comprehensive prevention services CC is a territorial indicator to differentiate its application areas and help preventing women from getting sick/dying from CC, highly preventable health problem.

The WHO indicates accessibility to health care as an international key objective in meeting the health needs of the population [19]. Medical care should be accessible and equitable for all people, no matter how much money they have, based on sustainable economic, social, and political attention.

Furthermore, the spatial dynamics of this illness (geography of health perspective) should be taken into account while also offering women access to CC education, vaccination and screening or in any given case, with treatment to improve their quality of life.

In our research, the GLMM provides a summary of the geographical pattern of CC incidence in SLP state that takes into account spatial variation in socio-economic factors and the accessibility to health services. Research indicates that recognising higher risk areas using the spatial model will help in targeting women to increase the efficiency of CCPS and different risk areas may have different screening intervals and even different screening tests [35]. Furthermore, GLMM allow recognising patterns of geographical distribution, patterns in small areas and/or little frequent pathologies. In addition, small area analysis tends to reduce ecological fallacy. This is consistent with previous literature that concluded that associations over a set of geographical areas do not necessarily imply that these factors apply to individuals [25, 26, 38, 48].

As previously advocated, it is necessary to integrate the geographic dynamics of this illness in formulating CCSP policies in Mexico [12, 49, 50]. CC is a preventable illness, since it is a late complication of a persistent infection by HPV and the final result of a chain of events, which may take years to develop to advanced stages.

## Conclusions

The results from the GLMM make evident an increase in relative risk (RR) in the incidence of CC, in areas with concentration of municipalities that have deficient socioeconomic conditions and low accessibility to services of prevention, early detection, diagnosis and management of CC. This relation varies throughout the territory of SLP state due to the low coverage of CCSP, high marginalisation, and the low accessibility to health services these being important factors in the explanation of spatial patterning of the RR to CC incidence. The geographic variation reflects differences in personal behaviours, local differences, and differences in screening uptake rates by location.

As mentioned in the discussion, the political standards that determine the characteristics (location, type, resources, etc.) of medical unit services are not made according to the needs of the population and the spatiality of CC.

The evolution of this type of cancer provides a very valuable time period to educate the population about how to prevent the infection of HPV such as a sexually transmitted disease to identify the illness at an early stage. Early detection of the sexually transmitted HPV infection is key to modifying or avoiding this CC risk factor. Furthermore, we find it valuable that the national CCSP takes into account the spatial dynamics of this illness while also offering women better education concerning vaccination, screening or for specific individuals how treatment can improve their quality of life.

Accordingly, from the results presented here, this investigation suggests:

- Considering the living conditions of women under a geographical view.
- In areas where screening is poor, HPV vaccination should be widespread.
- Thinking of these research results as guidelines for a subsequent health political action, with priority in areas with a concentration of municipalities with high risk.
- Creating a geo-referenced database system available at the individual level, which permits a spatial analysis at the individual level (i.e. individual socioeconomic data, behaviour and knowledge about prevention), plus is spatially organized and maximises the benefits of CCSP.

Ignoring the spatial variability means to continue a public policy that does not tackle deficiencies in its national CCSP and to keep disadvantaging and disempowering Mexican women in regard to their health care.

## Abbreviations

2SFCA: Two-step floating catchment area
ASR: Age standardised incidence rate
BGA: Basic geostatistical areas
C53: Malignant neoplasm of cervix uteri
CAR: Conditional autoregressive model
CC: Cervical cancer
CCSP: Cervical cancer screening programme
CHICAS: Combining Health information, computation and statistics
CI: Posterior 95 % credible interval
CIN3: Cervical intra-epithelial neoplasia grade III
CONAPO: National population council
CTS: Communications and transportation secretariat
D06: Carcinoma in situ of cérvix uteri
DIC: Deviance information criterion
GDP: Gross domestic product
GLMM: Generalised linear mixed models
HPV: Human papillomavirus
HR-HPV: High-risk human papillomavirus
IA: Index of accessibility to health services
ICD-10: International classification of diseases-10
INEGI: National Institute of Statistics and Geography
INLA: Integrated nested laplace approximations
ISCIII: Carlos III Institute of Health
MI: Marginalisation index
MU: Medical unit
NHS: National health service
OR: Odds ratio
PI: Positive screening index
PP: Posterior probabilities
RR: Relative risk
SF: Single female
SICAM: Mexican women´s cancer information system
SIR: Standardise incidence ratio
SLP: San Luis Potosí
SPSSA: Public service of the SSA
SSA: State health services
UAEM: Universidad Autónoma del Estado de México
UASLP: Universidad Autónoma de San Luis Potosí
UK: United Kingdom
UNAM: Universidad Nacional Autónoma de México
UNDP: United Nations Developments Programme
WHO: World Health Organization
